# Crop and varietal diversification of rainfed rice based cropping systems for higher productivity and profitability in Eastern India

**DOI:** 10.1371/journal.pone.0175709

**Published:** 2017-04-24

**Authors:** B. Lal, Priyanka Gautam, B. B. Panda, R. Raja, Teekam Singh, R. Tripathi, M. Shahid, A. K. Nayak

**Affiliations:** 1 Crop Production Division, ICAR-National Rice Research Institute, Cuttack, Odisha, India; 2 Regional Rainfed Lowland Rice Research Station (RRLRRS), Gerua, Assam, India; Louisiana State University College of Agriculture, UNITED STATES

## Abstract

Rice-rice system and rice fallows are no longer productive in Southeast Asia. Crop and varietal diversification of the rice based cropping systems may improve the productivity and profitability of the systems. Diversification is also a viable option to mitigate the risk of climate change. In Eastern India, farmers cultivate rice during rainy season (June–September) and land leftovers fallow after rice harvest in the post-rainy season (November–May) due to lack of sufficient rainfall or irrigation amenities. However, in lowland areas, sufficient residual soil moistures are available in rice fallow in the post-rainy season (November–March), which can be utilized for raising second crops in the region. Implementation of suitable crop/varietal diversification is thus very much vital to achieve this objective. To assess the yield performance of rice varieties under timely and late sown conditions and to evaluate the performance of dry season crops following them, three different duration rice cultivars were transplanted in July and August. In dry season several non-rice crops were sown in rice fallow to constitute a cropping system. The results revealed that tiller occurrence, biomass accumulation, dry matter remobilization, crop growth rate, and ultimately yield were significantly decreased under late transplanting. On an average, around 30% yield reduction obtained under late sowing may be due to low temperature stress and high rainfall at reproductive stages of the crop. Dry season crops following short duration rice cultivars performed better in terms of grain yield. In the dry season, toria was profitable when sown earlier and if sowing was delayed greengram was suitable. Highest system productivity and profitability under timely sown rice may be due to higher dry matter remobilization from source to sink. A significant correlation was observed between biomass production and grain yield. We infer that late transplanting decrease the tiller occurrence and assimilate remobilization efficiency, which may be responsible for the reduced grain yield.

## Introduction

Rice production in the tropics is sensitive to climatic factors such as temperature, rainfall, and solar radiation which affect the crop in various ways during different stages of its growth [1]. Climate change over the past few decades have been fairly rapid in many agricultural regions around the world, and increases in atmospheric carbon dioxide (CO_2_) and ozone (O_3_) levels have also been remarkable. Many studies confirm that significant changes have occurred in the climate of South East Asia during the 20^th^ Century [2–3] and that in some regions in the tropics, weather is already approaching critical levels during the susceptible stages of rice growth [4]. Thus, the observed climatic changes (and attributed weather fluctuations) in the past may have had significant influences on rice productivity in the region. Weather and climate affect plant growth and development, and the fluctuations and occurrences of climatic extremes particularly at critical crop growth stages may reduce yield significantly [5]. This makes climate variability a threat to food production leading to serious social and economic implications [6]. In South East Asia a clear understanding of the vulnerability of food crops as well as the agronomic impacts of climate variability enables one to implement adaptive strategies to mitigate its negative effects. Weather and climate variability influence the initiation of the rice cropping season since it is often synchronized with the onset of the rainy season.

In Asia, intensity of cropping in major rice ecosystems has been increased over the past four decades and spectacular production and yield increases have been achieved. However, there is growing evidence that even with the best available cultivars and agronomic management, factor productivity (i.e. yields at constant level of input) may be declining and that the long-term sustainability of these rice-based systems may be at risk [7–8]. The adverse impacts of climate change on rice production system have been increasing over recent years [9], necessitating urgent action [10]. Rice fallows may be the reason for constant productivity of rice, which basically imply to those lowland *kharif* sown rice areas which remain uncropped during *rabi* (winter) due to various reasons such as early withdrawal of monsoon rains leading to soil moisture stress at planting time of winter crops, waterlogging, lack of appropriate varieties of winter crops for late planting, and socio-economic problems like stray cattle, blue bulls etc. Rice fallows of South Asia accounts for 79% (11.65 m ha) of the total area (15.0 m ha) including India, Bangladesh and Pakistan [11]. The rice fallows have a great potential for cultivation of short-duration pulses and oilseeds. However, very little efforts has been made to efficiently utilize these rice fallow with appropriate technical and developmental back-up. It is expected that nearly 3.0 million hectare area of rice fallows can be brought under cultivation, which can provide about 1.5–2.0 million tonnes of additional food grain production [12], and help in meeting the increasing demands of pulses and oilseeds. Development and popularization of improved varieties of pulses and oilseeds suiting to rice fallows of different agro-ecological regions coupled with improved agro-technology will boost production, and thus improve income and livelihood security as well as nutritional security of farming community. Moreover, introduction of legumes can provide a sustainable production base to the continued rice mono-cropped system, which is otherwise leading to decline in total factor productivity. Crop growth, development, water use, and yield under normal conditions are largely determined by weather during the growing season. Even with minor deviations from the normal weather, the efficiency of applied inputs and food production is seriously impaired [13]. Most pronounced effect of climate change is a drastic change in the rainfall pattern in the form of delayed monsoon, early withdrawl or inadequate precipitation with poor distribution leading to either drought or waterlogged conditions, particularly in rainfed ecosystem. Therefore, timing of rice transplanting plays an important role in getting higher yield of rice and sowing of succeeding dry season crops mainly pulses or oilseeds.

The rainfed rice area constitutes 12.9 m ha in Eastern India including the states of Odisha, West Bengal, Jharkhand, Assam, Chhattisgarh, Bihar, which are characterized by soils, relatively heavier in texture; these soils have better moisture holding capacity to support a good second crops, these are mostly kept fallow after rice in most of the areas. During kharif season, rice is grown with traditional practices and consequently land become unavailable for the sowing of second crop when sufficient residual moisture is available. The land can be brought under double cropping with proper utilization of carry-over residual soil moisture. Keeping the importance of above facts in mind, the present study was conducted with the hypothesis that productivity and profitability of rainfed rice fallow systems can be improved by growing a second crop with residual soil moisture management. The study was undertaken in rainfed rice based cropping systems in Eastern India with the following objectives i) to explore the possibility of growing short duration pulse and oilseed crops after *kharif* rice crop ii) to test the effects of delayed transplanting on agronomic traits that are related to the grain yield of rice and succeeding dry season crops.

## Materials and methods

### Site description

Experiments were conducted during the wet and dry seasons of 2012 to 2014 at Central Rice Research Institute, Cuttack (20° 26^/^ N, 85° 56^/^ E; elevation 24 m above mean sea level). The two main cropping seasons at this site include a kharif or wet season from June to November and a rabi or dry season from December to April. The soil of the experimental site was an Aeric Endoaquept with sandy clay loam texture (30.9% clay, 16.6% silt, 52.5% sand), bulk density 1.40 Mg m^3^, pH (using 1:2.5, soil: water suspension) 6.7, electrical conductivity (EC) 0.078 dS m^-1^, available N, P and K- 223, 15.5, and 104 kg ha^-1^, respectively.

### Experimental set-up

**Fig 1:**
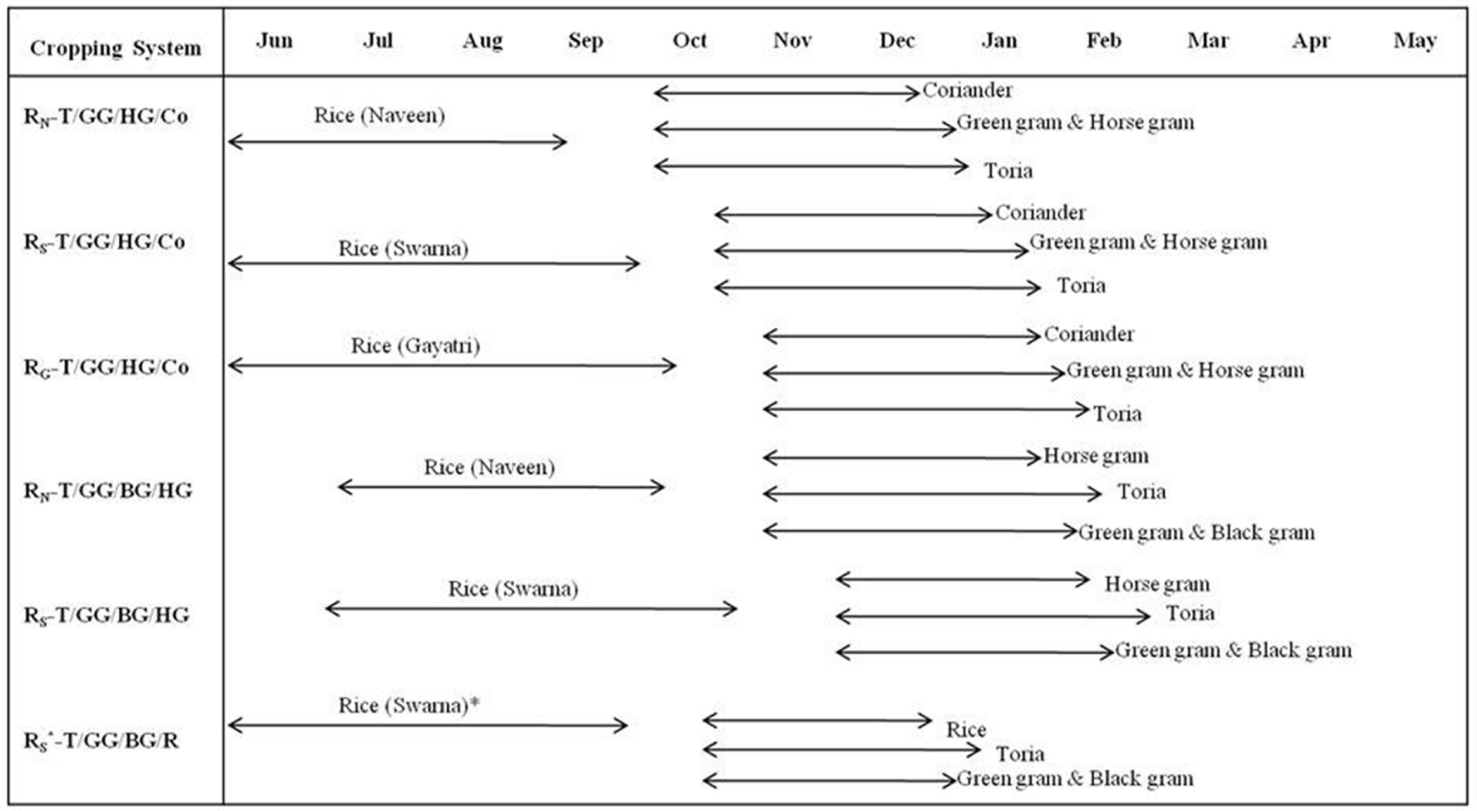
Crop calendar of the rice based cropping systems evaluated in the experiment. R: Rice; T: Toria; HG: Horse gram; Co: Coriander; GG: Green gram; BG: Black gram, letters in the subscript with rice (R) represents the rice cultivars, N: Naveen; S: Swarna; G: Gayatri; A: Annada. *In this system rice variety Swarna planted on first July was artificially damaged on 30^th^ Sept. assuming weather aberration.

In kharif season, three improved varieties of rice Naveen (N), Swarna (S) and Gayatri (G) with different durations i.e. 120, 145, and 160 days, respectively were transplanted during 1^st^ week of July and 1^st^ week of August following randomized block design. Of the three rice varieties, Gayatri and Swarna were photoperiod sensitive and Naveen was photoperiod insensitive. In the rabi season, kharif season plots were divided into four plots and different non-rice crops were sown viz., greengram (GG), horsegram (HG), toria (T), Coriander (Co), blackgram (BG) (Fig 1). The experiment was laid out with the following cropping systems: R_N_-GG/HG/T/Co, R_S_-GG/HG/T/Co, R_G_-GG/HG/T/Co, R_N_-GG/HG/T/BG, R_S_-GG/HG/T/BG and R_S_*-GG/HG/T/R in a randomized complete block design with 8.5 m x 6.5 m plots replicated thrice. In the treatment (R_S*_–R_A_/T/BG/GG) first crop of rice cv. Swarna sown on 1^st^ week of July was damaged artificially assuming drought/flood occurred due to weather aberrations, after that a short duration (95 days) rice variety Annada (A) and short duration pulse and oilseed crop was sown in October. The cropping system was framed, keeping in mind the conditions of rainfed lowlands, due to weather aberrations, there may be a heavy rainfall or a long dry spell in the mid-crop growth period. The same conditions did not happen in our experiment, but we assume such conditions and cut down the crop manually and ploughed down the field and within 15 days sowing of next crops was done short duration rice variety Annada was compared with dry crops from the same time in the mid-season to evaluate whether Annada rice or dry crops are more profitable and productive.

Field was prepared with a tractor drawn plough followed by puddling and laddering in kharif season for transplanting of rice. 25 days old rice seedlings for Naveen and 30 days old for Swarna and Gayatri were transplanted in 1st week of July and 1st week of August as per the treatments. For all dry season crops, field was prepared by dry ploughing and seeds were sown by hand. Nutrients were applied as per the recommended doses of fertilizer to all crops in the cropping systems (“S1 Appendix”). Rain was sufficient to fulfill the water needs in kharif season; therefore no irrigation was applied to rice. Rabi crops were irrigated with ground water whenever necessary, with about 50 mm water per irrigation. Insect pests and diseases infestation was below economic threshold except in toria. Chemical protection measures were taken against bacterial leaf blight in rice and aphids in toria during both the years. Details of input used in different individual crops are given in “S2 Appendix”.

### Growth characteristics

#### Phenology

After the rice seedlings were transplanted to the field, the numbers of tiller in10 fixed hills from each treatment were surveyed at 3-days intervals from 21 to 60 days in Naveen and 21 to 68 days in Swarna and Gayatri. Height of 5 randomly selected plants from the net plot was measured from the soil surface to the tip of the tallest leaf. However, after heading the height was measured upto the tip of rice panicle. The growth stage of rice i.e. maximum tillering (MT), panicle initiation (PI), booting, panicle emergence, flowering, and maturity in every treatment were recorded. From the day of tiller emergence, the tillers on tagged plants were counted every alternate day. The date on which 50% of the tagged plants in each treatment bear maximum tillers was considered as maximum tillering stage. For observing, PI stage, mother tiller was uprooted and a transverse cut was made at the base of the plant, a thread like appearance was seen with magnifying glass. This was observed on each alternate day for recording the PI stage. Days to flowering were determined when 90% of the hills in each treatment had at least one tiller that reached anthesis. Days to maturity were determined when 95% of the spikelets within each treatment had turned yellow.

#### Biomass accumulation and growth analysis

The above-ground parts of the rice plants from 5 hills from each treatment were sampled periodically over a month interval and taken to the laboratory, where they were dried at 80°C for 48 h till constant weight of the samples was observed and subsequently divided into three (leaf; stem and panicle at the flowering and maturity stages) parts. Then, the separated organs were dried at 70°C until constant weight and weighed to determine the dry matter weight. From the biomass of periodical interval crop growth rate (CGR) and relative growth rate (RGR) was calculated

$$CGR\;\left(gm^{-2}d^{-1}\right)=\frac{w_{2}-w_{1}}{t_{2}-\;t_{1}}$$

$$RGR\;\left(gg^{-1}d^{-1}\right)=\frac{lnw_{2}-lnw_{1}}{t_{2}-\;t_{1}}$$

Where w_1_ and w_2_ represent the dry matter weight of rice plants as measured during the first and second times, respectively, and the difference between t_2_ and t_1_ is the time interval of the two measurements.

Based on the parameters that were explicated above, the dry matter remobilization amount (DMRA), dry matter remobilization efficiency (DMRE) and dry matter conversion rate (DMCR), were calculated according to the following equations [14].

Dry matter remobilization amount(g)=dry matter weight per tiller at flowering−dry matter weight per tiller at maturity

Dry matter remobilization efficiency(%)=dry matter remobilizationamount/dry matter weight per tiller at maturity ×100

Dry matter conversion rate(%)=dry matter remobilizationamount/dry weight of the grains per tiller at maturity ×100

### Yield attributes and yield

At maturity, ten plants of each treatment were randomly selected to measure yield attributes (excluding the border plants). Number of panicles were counted from each treatment, and five panicles per plant were randomly selected for measuring panicle length, panicle weight, number of spikelets per panicle, spikelet fertility percentage (proportion of filled and chaff grains) and 1000 grain weight was determined. The rice crop was harvested and sun dried for 3 days, then total produce was weighed and recorded as total biomass. The produce was then threshed and grains were separated, dried (upto 14% moisture content) and weighed for grain yield. The rice grain yield was determined with the moisture content being adjusted to 14%. Similarly, yield of non-rice crops was also recorded.

Rice equivalent yield (REY) was calculated to compare system performance by converting the yield of non-rice crops into equivalent rice yield on a price basis, using the formula: REY = Yx(Px/Pr), where Yx is the yield of non-rice crops (kg ha^-1^), Px is the price of non-rice crops (Rs. kg^-1^), and Pr is the price of rice (Rs. kg^-1^). Prices of individual inputs and outputs were assumed to be stable during the experimental period. For calculating system productivity, rice yield of wet season and rice equivalent yield of dry season crops were summed up and expressed as kg ha^-1^.

### Economic analysis

Net return (INR ha^−1^) and benefit: cost (B: C) ratio were calculated by considering the sale prices of different wet and dry season crops and cost of cultivation. Economics based on the average data of the different crops was computed by using variable cost and income from sale of rice and dry season crops (grain and straw/stover). The variable cost of cultivation of rice and other crops included cost involved in different operations (eg. rice nursery raising, tillage for seed bed preparation, field preparation, seeding, weeding, harvesting, threshing), and the inputs (seed, irrigation, fertilizers, agro-chemicals, and labors) used for raising the crops. The cost of cultivation was kept same for both July and August sown crops as the inputs used are same. The economic analysis, however, does not include the value of land. Gross return was calculated by multiplying grain and straw yield with the price of grain and straw. Minimum support price (MSP) fixed by the government of rice (Rs. 13.6 kg^-1^), green gram (Rs. 46 kg^-1^), horse gram ((Rs. 38 kg^-1^), toria (Rs. 30.5 kg^-1^), coriander (Rs. 53 kg^-1^) and black gram (Rs. 43.5 kg^-1^) were used, whereas, the price of rice straw (Rs. 2.5 kg^-1^), stover of green gram, black gram and horse gram (Rs. 1 kg^-1^), toria (Rs. 3 kg^-1^) and coriander (Rs. 0.5 kg^-1^) were taken as the prevailing price in the local markets at the time of harvest. Net returns, B: C ratio, land use efficiency, production efficiency and economic efficiency were calculated by the following formulas:

$$Net\;returns\;\left(INR\;ha^{-1}\right)=Gross\;returns\;\left(INR\;ha^{-1}\right)-Cost\;of\;cultivation$$

$$B:C\;ratio=\;Net\;returns\;\left(INR\;ha^{-1}\right)/\;Cost\;of\;cultivation$$

Land use efficiency=Number of days land is used in a year × 100/365

$$Production\;efficiency\;\left(kg\;ha^{-1}day^{-1}\right)=\;grain\;yield\;\left(kg\;ha^{-1}\right)/total\;duration\;of\;crops\;\left(days\right)$$

$$Economic\;efficiency\;\left(Rs.\;ha^{-1}day^{-1}\right)=\;net\;returns\left(Rs.\;ha^{-1}\right)/total\;duration\;of\;crops\;\left(days\right)$$

### Statistical analysis

The data for all the parameters were analyzed by using SAS version 9.3 for a randomized complete block design. Data for growth and yield attributes, yield and economics were analyzed in ANOVA. Association between different parameters were studied using correlation and linear regression analysis. Statistical significance was set at an alpha level of 0.05. Means were compared by the least significant difference (LSD) test if the f-value was significant.

## Results

### Weather conditions

**Fig 2:**
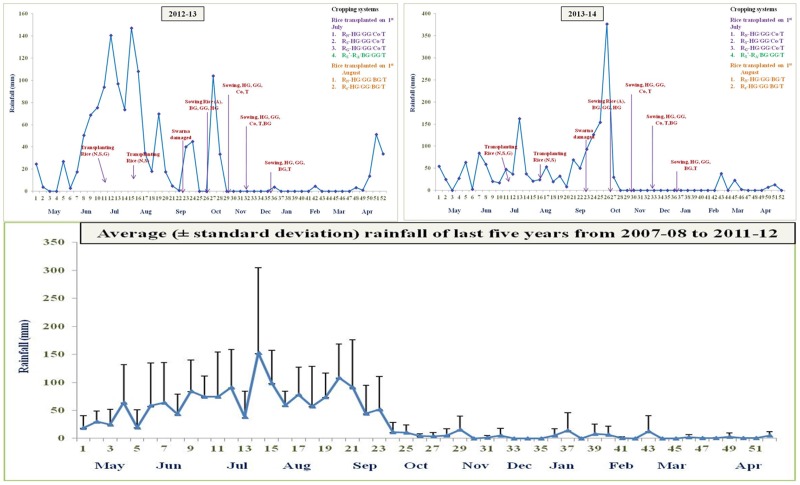
Rainfall received during experimentation and sowing of different crops of the rice based cropping systems evaluated in the experiment during 2012–13 (Fig 2a) and 2013–14 (Fig 2b). R: Rice; T: Toria; HG: Horse gram; Co: Coriander; GG: Green gram; BG: Black gram, letters in the subscript with rice (R) represents the rice cultivars, N: Naveen; S: Swarna; G: Gayatri; A: Annada. *In this system rice variety Swarna planted on first July was artificially damaged on 30^th^ Sept. assuming weather aberration. **Fig 2c.** Average rainfall (mm) received during last five years (from 2007–08 to 2011–12) before initiation of the experiment. Vertical bars on the line represent standard deviation of the five years.

The meteorological data showed a marked variation in weather conditions during the two years of the experiment. Maximum and minimum temperatures, evaporation, and sunshine hours remained almost constant during both the years, whereas, rainfall varied significantly. Rice and dry season crops received 1410 and 1777 mm rainfall during 2012–13 and 2013–14, respectively against average of 1622 mm rainfall during last five years (Fig 2). In the year 2013–14, cyclone *Phailin* also affected the crop growth. Due to cyclone around 699 mm rainfall was received during October 2013 which coincided with the reproductive stage of late sown/long duration rice varieties. Maximum temperature ranged from 27–37°C in 2012–13 and 27–40°C in 2013–14 with mean around 31°C during both the years (“S3 Appendix”). Minimum temperature also varied from 14–27°C in 2012–13 and 15–25°C in 2013–14 with mean around 22°C during both the years. Mean evaporation and sunshine hours in both the years was 5.7 mm and around 4 hours, respectively. The pattern of rainfall also varied during both the years. In 2012–13, 72.3% (1019.8 mm) of rainfall received during south-west monsoon period i.e. June to September, whereas, in 2013–14, it was only 46.4% (825.3 mm) (Fig 2c).

### Tiller dynamics

**Fig 3:**
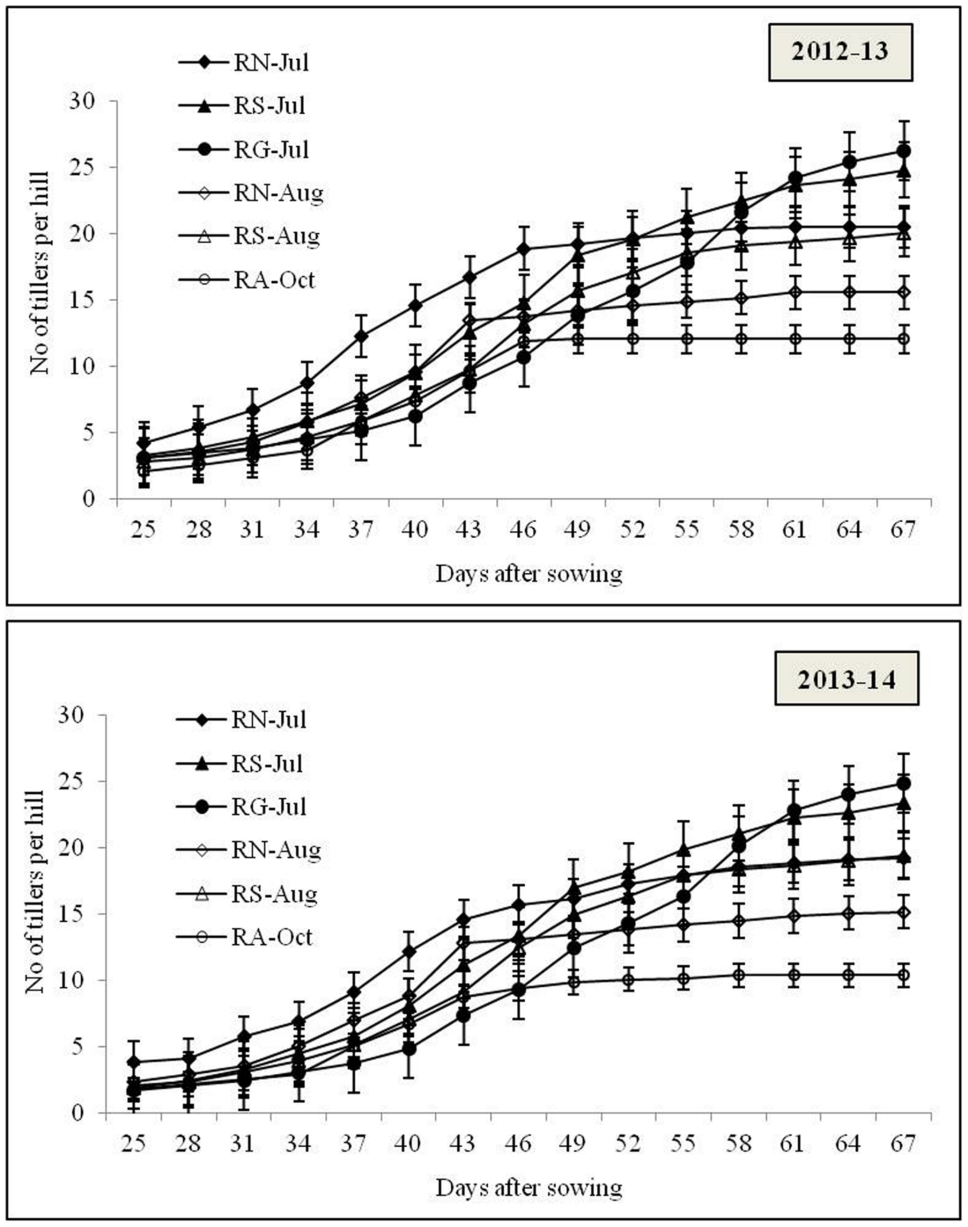
Dynamics of the tiller occurrence after 25 days of transplanting to maximum tillering stage of the rice varieties grown in *Kharif* season during 2012–13 and 2013–14. R: Rice; N: Naveen; S: Swarna; G: Gayatri; A: Annada.

The dynamics of occurrence of tillers in all the cultivars showed a distinct pattern, duration of cultivars and time of transplanting significantly influenced the tiller occurrence (Fig 3). Long duration cultivars showed higher number of tillers but commencement of maximum tillering stage was comparatively late compared to short duration varieties. Apart from that, timely transplanting in July significantly resulted in higher number of tillers per hill whereas, August transplanting decrease the tiller number up to 27.8 and 20.6% in 2012–13 and 2013–14, respectively. However, number of tillers per hill was lower in 2013–14 compared to preceding years.

### Dry matter accumulation and remobilization

**Fig 4:**
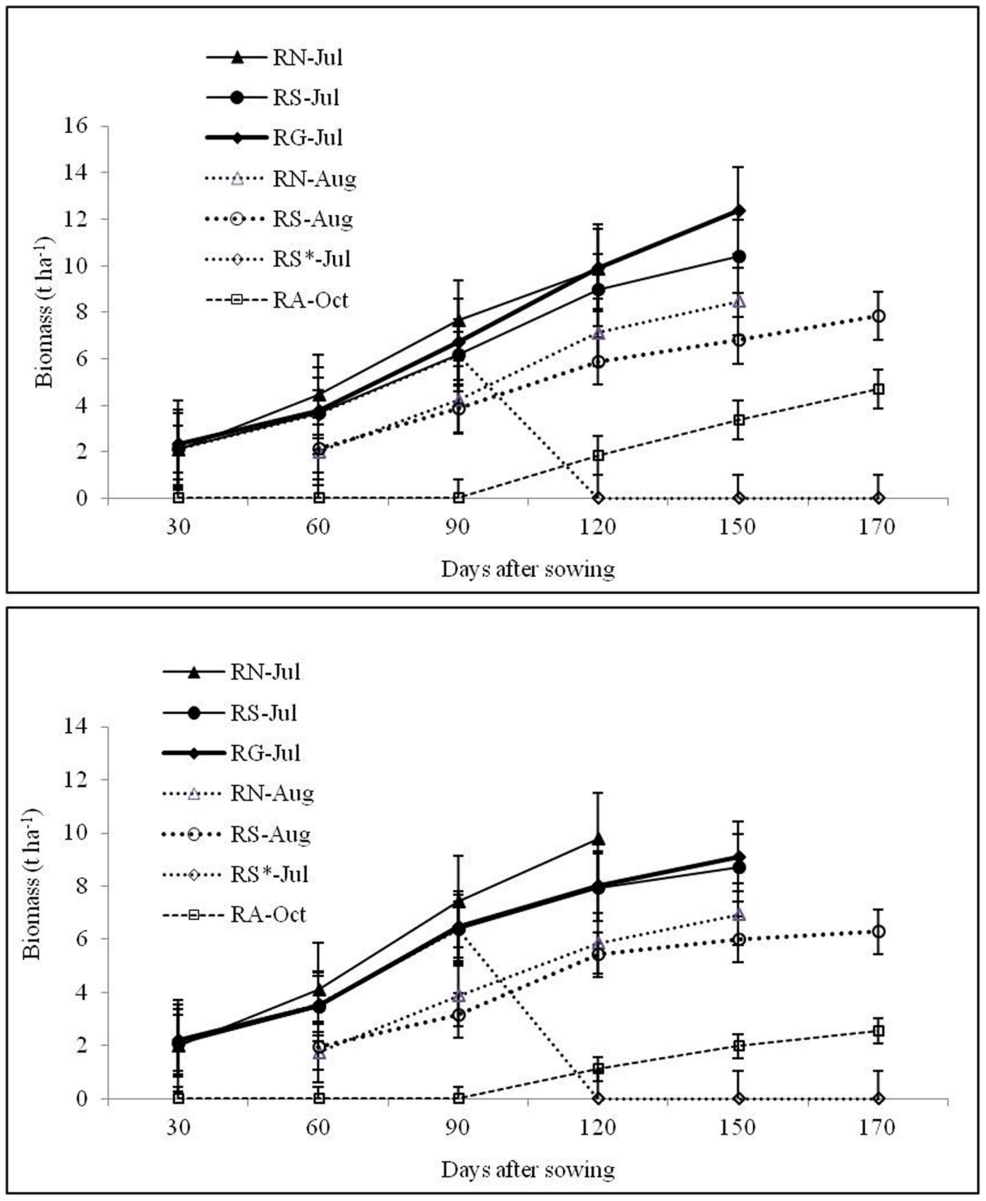
Biomass (t ha^-1^) recorded at periodical intervals of the rice varieties grown in *Kharif* season during 2012–13 and 2013–14. R: Rice; N: Naveen; S: Swarna; G: Gayatri; A: Annada. *In this system rice variety Swarna planted on first July was artificially damaged on 30^th^ Sept. assuming weather aberration.

Overall performance of the crops was better during 2012-13. The dry weight of above ground parts of rice was greater in long-duration varieties and rice transplanted in July yielded higher biomass compared to august transplanted rice during both the years (Fig 4). On an average, July transplanted rice produced 14.5 and 25.7% higher biomass over august transplanting during 2012–13 and 2013–14, respectively. In R_S_*-GG/HG/T/R cropping system, first crop of rice was artificially damaged assuming weather aberrations, so it contributed to biomass up to 90 DAS (days after sowing), after that second crop of rice (Annada) was sown and contributed to biomass up to harvest. Dry matter remobilization amount, efficiency, and contribution to the grain yield were highest for Gayatri followed by Swarna during both the years (Table 1). However, the amount, efficiency, and contribution were relatively low in the succeeding years, irrespective of transplanting date or cultivars. August transplanting resulted in decrease of DMRA, DMRE, and DMCR by 35.1, 5.5, and 7.3% in 2012–13 and 42.4, 6.2, and 9.1% in 2013–14, respectively. October sown Annada crop had significantly lowest remobilization of dry matter either from leaf, stem, or panicle during both flowering and maturity stage.

**Table 1:** Aboveground part dry matter weight (g), dry matter remobilization amount (DMRA in g), efficiency (DMRE in %) and conversion rate (DMCR in %) of rice at flowering and maturity stage.

Cultivars	Transplanting time	Flowering stage				Maturity stage						
		Leaf	Stem	Panicle	Total	Leaf	Stem	Panicle	Total	DMRA (g)	DMRE (%)	DMCR (%)
**2012–13**												
Naveen	July	0.665b	0.811c	0.291bc	1.767b	0.374bc	0.726c	1.716c	2.816c	1.049c	37.25b	61.13ab
Swarna		0.782a	0.968b	0.367a	2.117a	0.413b	0.889b	2.179b	3.481b	1.364b	39.18a	62.60a
Gayatri		0.896a	1.212a	0.416a	2.524a	0.521a	1.136a	2.368a	4.025a	1.501a	37.29b	63.39a
Naveen	August	0.611b	0.768d	0.266c	1.645c	0.312c	0.658d	1.632c	2.602c	0.957d	36.78b	58.64bc
Swarna		0.672b	0.849c	0.315b	1.836b	0.371bc	0.735c	1.721c	2.827c	0.991d	35.05c	57.58c
Annada	October	0.442c	0.625e	0.198d	1.265d	0.206d	0.326e	1.421d	1.953d	0.688e	35.23c	48.42d
**2013–14**												
Naveen	July	0.632b	0.764c	0.267bc	1.663b	0.336c	0.629c	1.659b	2.624b	0.961c	36.62b	57.93c
Swarna		0.768a	0.935b	0.359a	2.062a	0.398b	0.812b	2.112a	3.322a	1.264b	37.93a	59.66b
Gayatri		0.874a	1.168a	0.389a	2.431a	0.487a	1.098a	2.238a	3.823a	1.392a	36.41b	62.20a
Naveen	August	0.524c	0.639d	0.232c	1.395c	0.278d	0.438d	1.476c	2.192c	0.797d	36.36b	54.00d
Swarna		0.612c	0.771c	0.301b	1.684b	0.342c	0.642c	1.611b	2.595b	0.911c	35.11c	56.55c
Annada	October	0.398d	0.561e	0.175d	1.134d	0.184e	0.286e	1.215d	1.685d	0.551e	32.70d	45.35e

### Growth rate and growth stage

**Fig 5:**
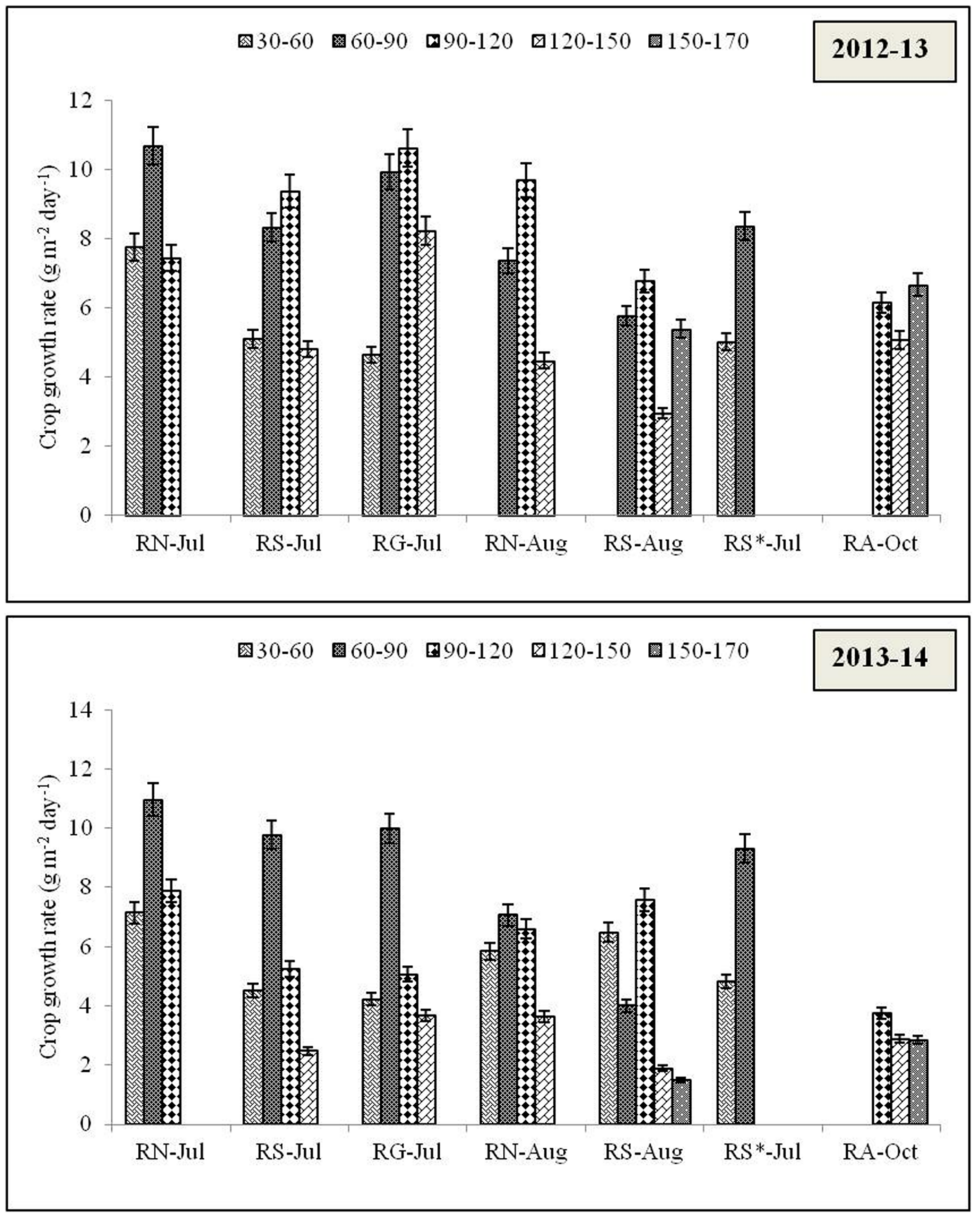
Crop growth rate (g m^-2^ day^-1^) recorded at periodical intervals of the rice varieties grown in *Kharif* season during 2012–13 and 2013–14. R: Rice; N: Naveen; S: Swarna; G: Gayatri; A: Annada. *In this system rice variety Swarna planted on first July was artificially damaged on 30^th^ Sept. assuming weather aberration.

**Fig 6:**
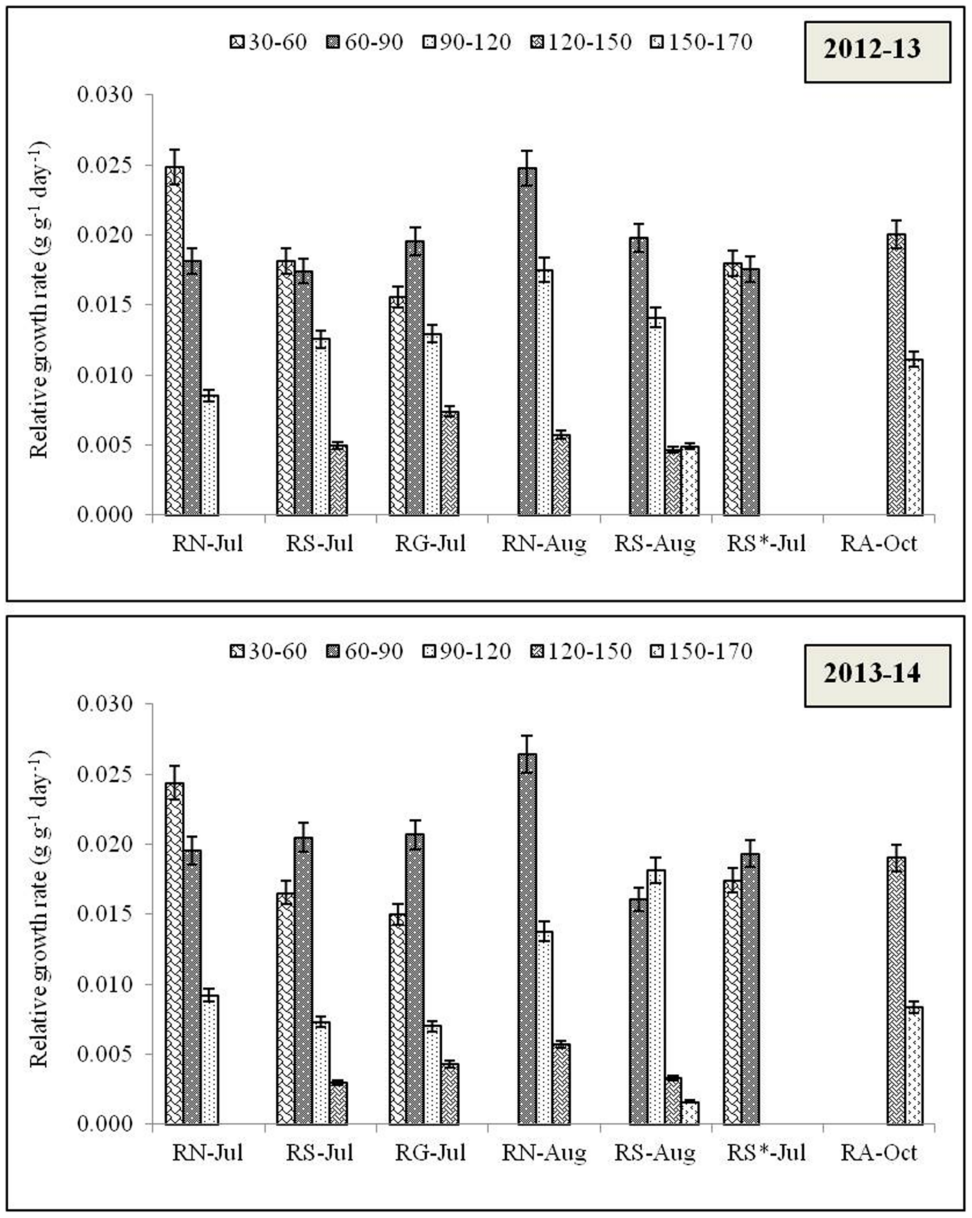
Relative growth rate (g g^-1^ day^-1^) recorded at periodical intervals of the rice varieties grown in *Kharif* season during 2012–13 and 2013–14. R: Rice; N: Naveen; S: Swarna; G: Gayatri; A: Annada. *In this system rice variety Swarna planted on first July was artificially damaged on 30^th^ Sept. assuming weather aberration.

Similar to biomass accumulation, crop growth and relative growth rate was greater in 2012–13 compared to 2013–14. Initial growth was slow in all the rice cultivars, in Naveen, highest growth rate was observed during 60–90 DAS whereas, in Swarna and Gayatri, it was higher during 90–120 DAS. During 2013–14, crop growth of July sown crops was better during 60–90 DAS but after that growth was considerably decreased (Fig 5). As per relative growth rate concerns, in short duration cultivars, it was relatively higher in 30–60 DAS whereas, in long duration cultivars, it was higher or at par in 60–90 DAS during both the years (Fig 6). The tillering, panicle initiation (PI), booting, panicle emergence (PE), flowering, and maturity stages of different rice cultivars were accounted in the experiment and it was found that late transplanting led to commencement of growth stages earlier than normal transplanting time during both the years. On an average, tillering, PI, and flowering stages occurred 7d, 6.5d, and 9d earlier in 2012–13 and 6d, 7d, and 9.5d earlier in 2013–14 due to late transplanting in August (Table 2).

**Table 2:** Number of days taken for different growth stages of rice during 2012–13 and 2013–14.

Cultivars	Transplanting	Tillering	PI	Booting	Panicle Emergence	Flowering	Maturity
**2012–13**							
Naveen	July	49	57	66	75	90	119
Swarna		61	81	89	97	115	143
Gayatri		65	89	98	108	128	157
Naveen	August	44	51	58	69	80	108
Swarna		52	78	85	91	110	136
**2013–14**							
Naveen	July	46	55	61	76	89	117
Swarna		58	85	92	101	121	146
Gayatri		64	93	101	115	130	161
Naveen	August	41	49	55	75	83	111
Swarna		51	75	83	92	108	135

### Yield attributes of rice

Production of yield attributes was influenced by transplanting dates, although varietal difference was also evident (Table 3). Rice cultivar Gayatri produced highest number of effective tillers, spikelets per panicle, and panicle weight followed by Swarna. Naveen and Swarna transplanted in July had significant differences in the production of tillers, panicles, spikelets, and fertility percentage over their transplanting in august. Effect of year was also significant which may be due to changing weather conditions. Production of all the yield attributes was decreased during 2013–14 except august transplanted Swarna in which it was increased. The difference in plant height and 1000- grain weight was not significant.

**Table 3:** Yield attributes of rice varieties grown during *Kharif* seasons of 2012–13 and 2013–14.

Cultivars	Sowing time	Plant height (cm)	No. of tillers m^-2^	No. of panicles plant^-1^	Spikelets panicle^-1^	Fertility %age	Panicle weight (g)	1000 grain weight (g)
**2012–13**								
Naveen	1^st^ week of Jul	116.4a	351c	10.15c	102.3c	84.1a	2.18b	21.51a
Swarna	1^st^ week of Jul	93.5b	435b	12.97b	118.4b	84.7a	2.34a	19.23a
Gayatri	1^st^ week of Jul	97.6b	496a	14.76a	129.8a	85.0aa	2.47a	22.22a
Naveen	1^st^ week of Aug	111.9a	289d	8.42d	97.8c	75.5b	2.01c	21.33a
Swarna	1^st^ week of Aug	82.8c	275d	8.15d	101.8c	72.5b	2.16b	19.09a
Swarna*	1^st^ week of Jul	-	-	-	-	-	-	-
Annada	1^st^ week of Oct	78.7c	241e	7.06e	91.40d	69.4c	1.96c	20.81a
**2013–14**								
Naveen	1^st^ week of Jul	115.9a	348c	10.02b	102.5b	84.7a	2.15a	21.32a
Swarna	1^st^ week of Jul	91.1c	403b	10.62b	105.3b	79.5a	2.09b	19.01a
Gayatri	1^st^ week of Jul	95.2c	415a	11.86a	110.2a	81.2a	2.13a	22.03a
Naveen	1^st^ week of Aug	105.6b	256e	7.12d	89.6c	71.4b	1.89bc	20.89a
Swarna	1^st^ week of Aug	84.6d	289d	9.63c	112.7a	79.3a	2.26a	19.21a
Swarna*	1^st^ week of Jul	-	-	-	-	-	-	-
Annada	1^st^ week of Oct	71.6e	212f	5.46e	78.9d	61.8b	1.76c	20.11a

*In this system rice variety Swarna planted on first July was artificially damaged on 30th Sept. assuming weather aberration.

### Grain yield of rice

**Fig 7:**
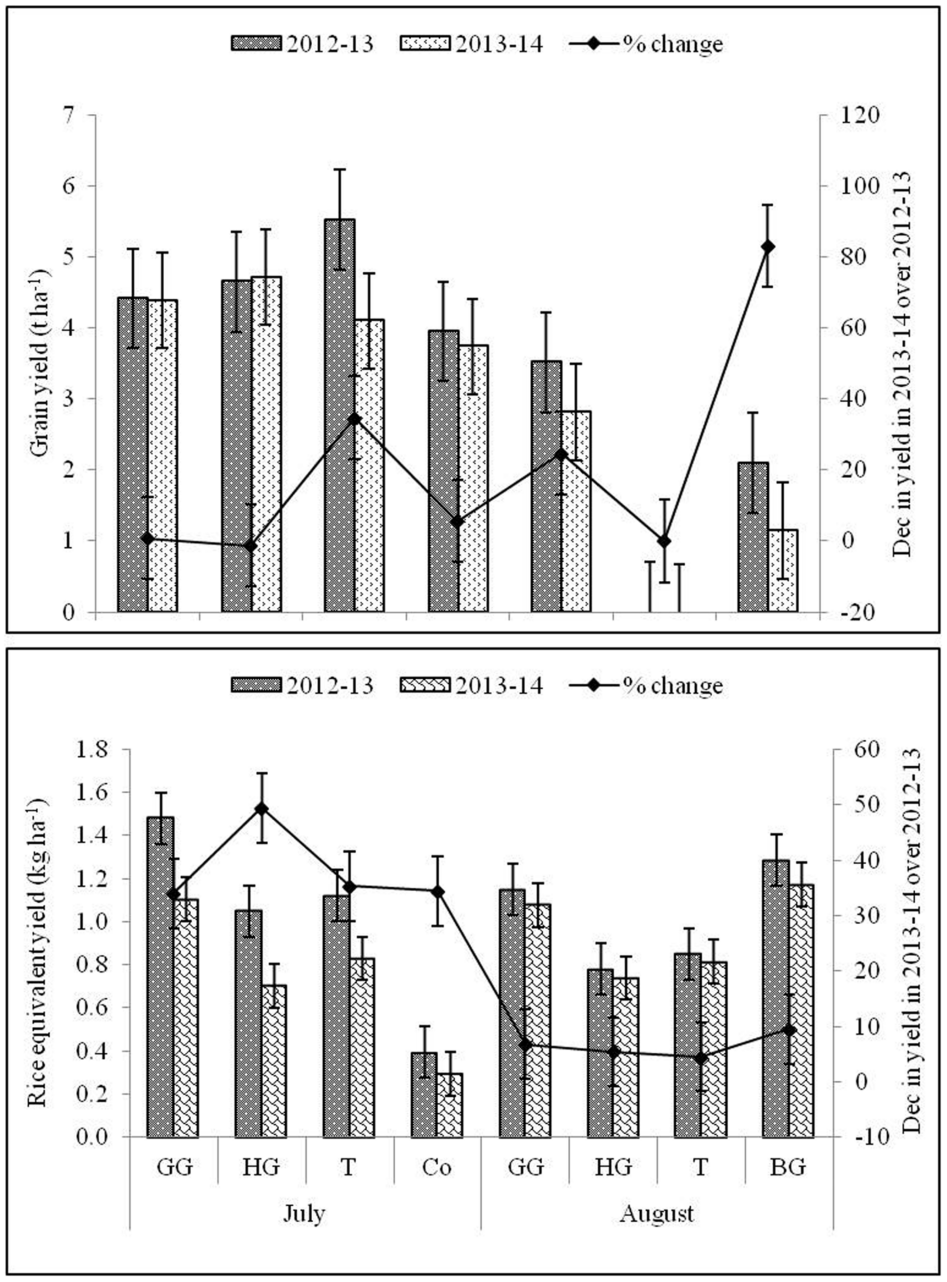
Grain yield of the rice varieties grown in *Kharif* season during 2012–13 and 2013–14 and rice equivalent yield of non-rice crops sown after July and August sown Naveen and Swarna. R: Rice; T: Toria; HG: Horse gram; Co: Coriander; GG: Green gram; BG: Black gram, N: Naveen; S: Swarna; G: Gayatri; A: Annada. *In this system rice variety Swarna planted on first July was artificially damaged on 30^th^ Sept. assuming weather aberration. Secondary axis represents the % change in yield during 2013–14 over 2012–13.

**Fig 8:**
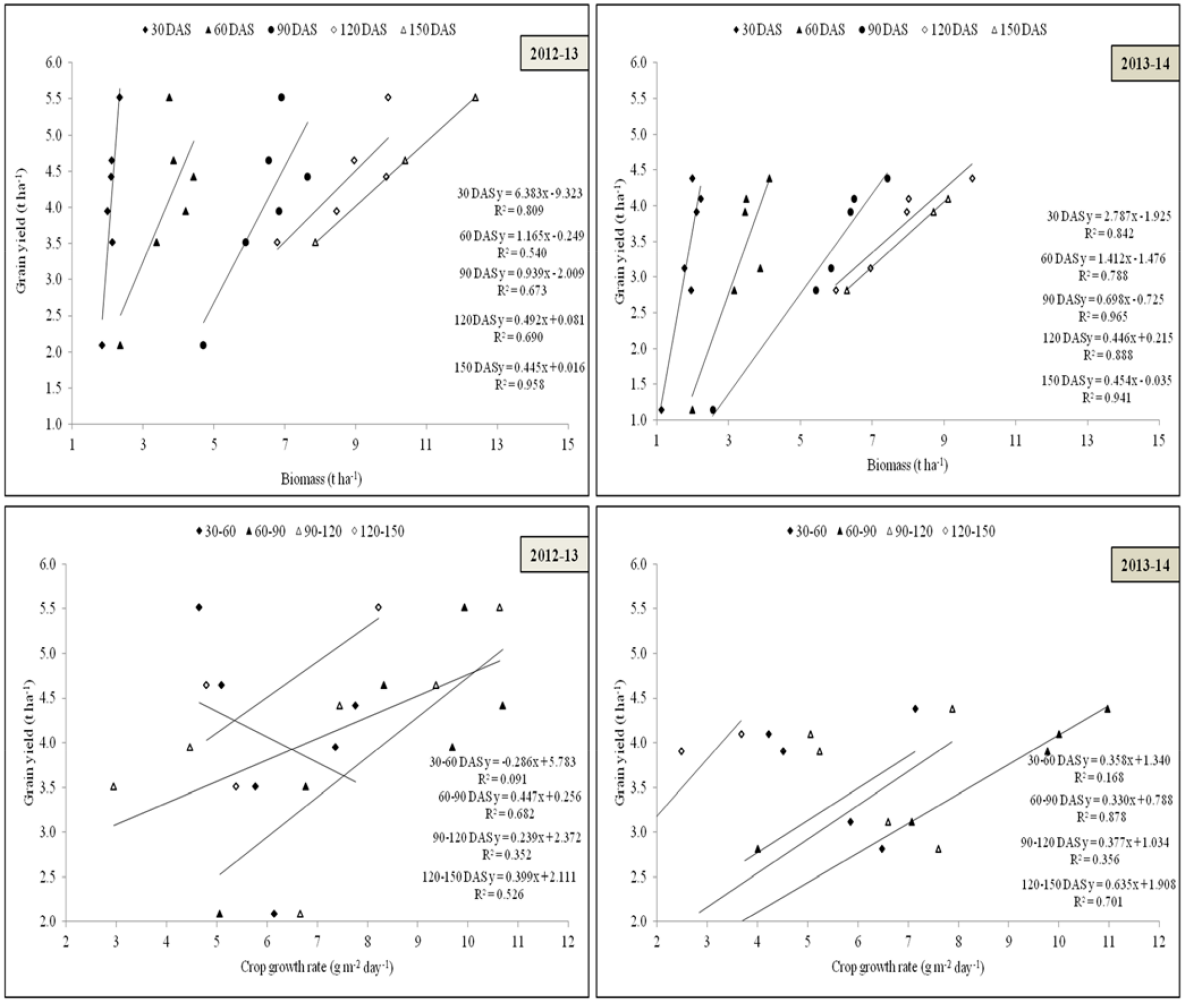
Relationship between grain yield and dry matter and crop growth rate at periodical intervals during 2012–13 and 2013–14.

**Fig 9:**
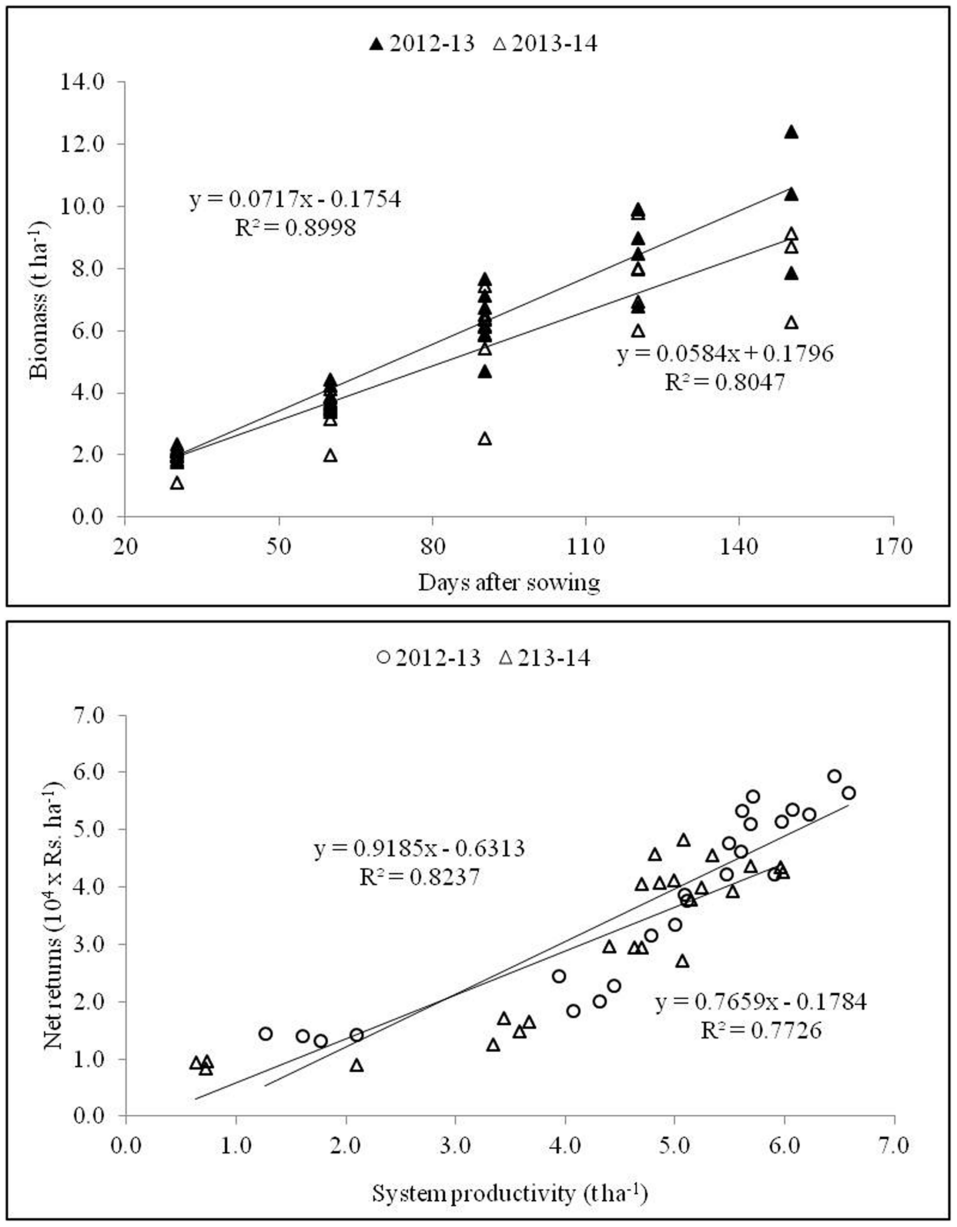
Correlations between: a) biomass (t ha^-1^) and days after sowing; b) system productivity (t ha^-1^) and net returns (Rs. ha^-1^) during 2012–13 and 2013–14.

The most important parameter i.e. yield which was affected significantly with different cultivars as well as transplanting date during both the years. Gayatri cultivars produced maximum rice yield (5.5 t ha^-1^) in 2012–13 and Swarna (4.7 t ha^-1^) in 2013–14 (Fig 7). Rice yield averaged across all the genotypes maximum (4.6 t ha^-1^) in rice which was 32.2% higher over August transplanting. Both the years of experimentation varied among themselves with respect to grain yield, and the yield was decreased in 2013–14 with more effect on Annada and Gayatri cultivars (Table 4). A positive correlation among grain yield, biomass and crop growth rate (Fig 8) and between biomass and days after sowing (Fig 9a) supported the findings.

**Table 4:** Economic yield of individual crops and rice equivalent yield of non-rice crops during 2012–13 and 2013–14.

S.No.	Rice cultivar and sowing time	Dry season crops	Grain yield (kg ha^-1^)				REY (kg ha^-1^)	
			Rice		Dry season crops			
			2012–13	2013–14	2012–13	2013–14	2012–13	2013–14
1	Naveen-1st July	Green gram	4420	4387	467	287	1545	950
2		Horse gram			413	231	1063	594
3		Toria			537	309	1185	682
4		Coriander			91	62	355	242
5	Swarna-1st July	Green gram	4651	4713	428	381	1416	1261
6		Horse gram			403	315	1037	811
7		Toria			479	442	1057	975
8		Coriander			112	89	436	347
9	Gayatri-1st July	Green gram	5526	4102	319	559	1056	1850
10		Horse gram			270	230	695	592
11		Toria			418	323	922	713
12		Coriander			97	75	378	292
13	Naveen-1st August	Black gram	3953	3741	513	468	1641	1497
14		Green gram			456	422	1509	1396
15		Toria			522	504	1151	1112
16		Horse gram			407	368	1047	947
17	Swarna-1st August	Black gram	3515	2818	290	265	928	848
18		Green gram			239	229	791	758
19		Toria			192	278	424	613
20		Horse gram			214	201	551	517
21	Swarna-1st July*	Black gram	-	-	550	228	1759	729
22		Green gram			483	217	1598	718
23		Toria			571	285	1260	629
24		Rice			2097	1145	2097	2097
	**LSD**_**p = 0.05**_				**171.55**	**179.43**	**305.52**	**311.52**

*In this system rice variety Swarna planted on first July was artificially damaged on 30th Sept. assuming weather aberration.

### Performance of dry-season crops and rice equivalent yield

Crop yields of non-rice crops in the individual cropping systems varied by climatic seasons and within the same climatic season, crop yields were much affected by the previous season rice cultivar grown. During both the years, seed yield of toria was significantly highest among all the dry season crops except when sown after Swarna (August) in 2012–13 and Gayatri (July) in 2013–14. Among all the crops, coriander recorded significantly lowest seed yield irrespective of the rice cultivar followed and years (Table 4). Overall, seed yield of dry season crops was higher in 2012–13 compared to 2013–14 except yield of green gram sown after Swarna (July) and toria sown after Swarna (August). Among crops, grown after July transplanted rice, toria proved its superiority in terms of seed yield followed by green gram. The performance of dry season crops was better when sown after Naveen except coriander irrespective of the time of transplanting.

Rice equivalent yield (REY) of dry-season crops was higher after Naveen, and July transplanting of rice cultivars was more favorable. Climatic conditions of 2013–14 years was affected the production of dry-season crops along with rice (Table 4). Regardless of the cultivar followed, REY of green gram was highest when followed by July transplanted rice, whereas, REY of blackgram was significantly highest when grown after August transplanted rice and damaged Swarna during both the years of experimentation. Black gram contributed to highest REY sown after damaged Swarna crop and late sown rice crops, it can be an option where crop failure can be minimized by sowing such crops. Although yield of toria was highest among all the dry season crops but its REY was lower than green gram and black gram. Similar to seed yield, REY of coriander was lowest among all the dry season crops irrespective of the cultivar followed and time of transplanting. As reflected in the seed yields, REY was higher in 2012–13 compared to 2013–14 except REY of green gram sown after Gayatri (July) and toria sown after Swarna (August). In R_S_*-BG/GG/T/R cropping system, first crop did not contributed to yield but early sowing of short duration rice contributed to maximum yield among all the crops irrespective of cropping systems during both the years. In this treatment, after rice, the seed yield of toria was highest during both the years followed by black gram but the REY was highest for black gram followed by green gram during both the years.

### System productivity and economic analysis

Both system productivity and annual net return must be considered for choosing suitable cropping systems under changing climatic scenario (Table 5). Among the cropping systems, Gayatri based cropping systems were more productive and profitable under normal weather conditions of 2012–13 followed by Swarna based systems but when climate changes as in 2013–14 the Swarna based cropping system were more profitable followed by Gayatri especially when transplanted in July. In August transplanting, Naveen based systems performed better during both the years. System productivity was highest for R_G_-GG (6.58 t ha^-1^) cropping system followed by R_G_-T, but net returns and B: C ratio was highest for R_G_-T followed by R_G_-GG. The results were supported by a positive correlation between net returns and system productivity during both the years (Fig 9b). Apart from rice crops, different dry season crops also contributed to the system productivity and profitability. Green gram contributed to the highest system productivity during both the years and productivity remained low where coriander was grown. This was also reflected in lower net return and B: C ratio of cropping system having coriander crop. The difference in productivity of green gram and horse gram was higher but the gainin net returns was not much visible, this may be due to the lower cost of production involved in horse gram. However, production and economic efficiency were significantly highest for Naveen either sown in July or August during both the years. Long duration cultivar had higher land use efficiency as compared to Naveen, which was around 15.3% higher (Table 6). Early transplanting of rice crops led to better productivity of subsequent crops leading to highest profitability compared to late transplanted rice. The R_S_*-BG/GG/T/R cropping system was not profitable and productive as other systems but it was a mid-season correction/contingent plan for mitigating the weather aberrations.

**Table 5:** System productivity and economics of the cropping systems followed in experiment during 2012–13 and 2013–14.

S.No.	Rice cultivar and sowing time	Dry season crops	System Productivity (kg ha^-1^)		Net returns (Rs. ha^-1^)		B:C ratio	
			2012–13	2013–14	2012–13	2013–14	2012–13	2013–14
1	Naveen-1st July	Green gram	5965	5337	51486	45771	1.99	1.88
2		Horse gram	5483	4981	47860	41204	1.97	1.84
3		Toria	5605	5069	53478	48332	2.04	1.94
4		Coriander	4775	4629	31697	29574	1.64	1.60
5	Swarna-1st July	Green gram	6067	5974	53678	42804	2.03	1.82
6		Horse gram	5688	5524	51156	39355	2.04	1.80
7		Toria	5708	5688	55897	43865	2.08	1.85
8		Coriander	5087	5060	38754	27195	1.79	1.55
9	Gayatri-1st July	Green gram	6582	5952	56456	43507	2.09	1.84
10		Horse gram	6221	4694	52789	40754	2.07	1.83
11		Toria	6448	4815	59531	45783	2.15	1.89
12		Coriander	5904	4394	42395	29737	1.86	1.60
13	Naveen-1st August	Black gram	5594	5238	46218	39961	1.89	1.77
14		Green gram	5462	5137	42282	37941	1.81	1.73
15		Toria	5104	4853	37678	40882	1.73	1.79
16		Horse gram	5000	4688	33456	29656	1.68	1.60
17	Swarna-1st August	Black gram	4443	3666	22833	16646	1.44	1.32
18		Green gram	4306	3576	20131	14863	1.39	1.29
19		Toria	3939	3431	24591	17296	1.48	1.34
20		Horse gram	4066	3335	18456	12668	1.37	1.26
21	Swarna-1st July*	Black gram	1759	729	13176	9812	1.46	1.34
22		Green gram	1598	718	14158	8515	1.50	1.30
23		Toria	1260	629	14487	9545	1.47	1.31
24		Rice	2097	2097	14405	9050	1.43	1.27
	**LSD**_**p = 0.05**_		**658.9**	**704.6**	**2115.6**	**2453.2**	**0.784**	**0.942**

*In this system rice variety Swarna planted on first July was artificially damaged on 30th Sept. assuming weather aberration.

**Table 6:** Production, economic and land use efficiency of the cropping systems followed in experiment during 2012–13 and 2013–14.

S.No.	Rice cultivar and sowing time	Dry season crops	Production efficiency (kg ha^-1^ d^-1^)		Economic efficiency (Rs. ha^-1^ d^-1^)		Land use efficiency
			2012–13	2013–14	2012–13	2013–14	
1	Naveen-1st July	Green gram	27.1	24.3	234.0	208.1	60.3
2		Horse gram	27.4	24.9	239.3	206.0	54.8
3		Toria	24.4	22.0	232.5	210.1	63.0
4		Coriander	22.7	22.0	150.9	140.8	57.5
5	Swarna-1st July	Green gram	24.8	24.4	219.1	174.7	67.1
6		Horse gram	25.3	24.5	227.4	174.9	61.6
7		Toria	22.4	22.3	219.2	172.0	69.9
8		Coriander	21.6	21.5	164.9	115.7	64.4
9	Gayatri-1st July	Green gram	25.3	22.9	217.1	167.3	71.2
10		Horse gram	25.9	19.6	220.0	169.8	65.8
11		Toria	23.9	17.8	220.5	169.6	74.0
12		Coriander	23.6	17.6	169.6	118.9	68.5
13	Naveen-1st August	Black gram	26.4	24.7	218.0	188.5	58.1
14		Green gram	25.8	24.2	199.4	179.0	58.1
15		Toria	23.0	21.9	169.7	184.2	60.8
16		Horse gram	26.0	24.4	174.3	154.5	52.6
17	Swarna-1st August	Black gram	18.7	15.4	95.9	69.9	65.2
18		Green gram	18.1	15.0	84.6	62.4	65.2
19		Toria	15.9	13.8	99.2	69.7	67.9
20		Horse gram	18.7	15.3	84.7	58.1	59.7
21	Swarna-1st July*	Black gram	8.8	3.6	65.9	49.1	54.8
22		Green gram	8.0	3.6	70.8	42.6	54.8
23		Toria	6.0	3.0	69.0	45.5	57.5
24		Rice	10.2	10.2	69.9	43.9	56.4
	**LSD**_**p = 0.05**_	**-**	**7.89**	**9.54**	**32.45**	**41.81**	**11.23**

*In this system rice variety Swarna planted on first July was artificially damaged on 30th Sept. assuming weather aberration.

## Discussion

The performance of all the crops in the cropping system in terms of their grain and biomass production varied considerably between cultivars and years. The productivity of different cropping systems was in the following order: R_G_-GG>R_G_-T >R_G_-HG>R_S_-GG>R_N_-GG (*P = 0*.*05*). Climate change will have varying impacts on cropping systems around the world, due to regional differences in rates of daytime and nighttime warming, changes to the timing, frequency, and intensity of P, and exposure to O_3_ and air pollution sources. About 78.7% of rice area in the region is rainfed where rice is grown only during rainy season (June–September). Though a major portion (48%), particularly lowland rice areas, is able to support a good second crop with carry-over residual soil moisture (due to heavy texture and high moisture retention), it is mostly mono-cropped. Inclusion of short duration low water requiring legumes (grain/green manure purpose) offered excellent opportunity to utilize carry-over residual soil moisture in rice fallow [15]. Crop/varietal diversification has been recognized as an effective adaptation option for farmers for risk mitigation [16]. Crop diversification has often been examined as a tool to stabilize crop revenue and farm income [17–18]. The exceptional rainfall in October, 2013 provided circumstantial evidence with regard to difference in productivity among both the years. Long duration rice cultivars were strongly affected, and the crops following them were also affected. It is clear that rainfall around the sowing period is a major factor determining the water contents of the soil and hence the success of crop establishment. In rice, leaf areas, leaf shapes to maximize photosynthetic efficiency, leaf area index (LAI) at flowering and crop growth rate (CGR) during panicle initiation well-developed root systems, have been identified as the major determinants of yield [19]. Apart from climate, among the crop production tools, proper time and method of sowing are the prerequisites that allow the crops to complete its life phase timely and successfully under a specific agro-ecology. Among the different components of agronomic packages for rice cultivation, the date of transplanting is one of the important factors as early or late transplanting may face different types of abiotic stress [20]. It is also crucial for successful dry season cropping following rice especially if conditions are dependent on rains,particularly at the end of the rainy season or the beginning of the dry season, the temporal variability within each site associated with rainfall could mask this trend. The optimum window for sowing is generally rather narrow, and will be determined by the interaction between crop growth and the prevailing environmental conditions. For successful rice production, timely planting, suitable transplanting densities and proper water, fertilizer and pest management are essential for improving the growth variables responsible for high yield [21]. In this study timely planting (July) resulted in higher biomass accumulation, yield and productivity. The superiority of this planting date can be explained by the ideal temperature, rainfall and higher dry matter allocation to the panicle and the extension of this process until the harvest time.

Planting date is more dependent on climatic conditions compared to other aspects of agricultural management. In certain periods of rice growth, dry matter accumulation in plant is larger than its consumption level for growth. In this state, excess photosynthetic materials are mainly gathered in the stem, and in the later stages of growth, which normally starts 2 or 3 weeks after the flowering stage, they are transferred to the grain via the remobilization process [22–23]. One of the effective factors on the remobilization rate is the source to sink ratio; the high and low levels of this ratio would result in the increase and decrease of remobilization respectively [24]. According to Yang *et al*. [24] despite the accumulation of abundant biomass in vegetative organs in some rice cultivars, these crops were not capable of using the respective matters at the end of the growth stage, which led to yield reduction and decline in the harvest index in these cultivars. According to Kirchhof*et al*. [25], in Philippines, the risk of damage Typhoon may also reduced by early sowing of rice followed by subsequent sowing of mungbean.

Phenological growth stages of rice were also varied with time, because rice plants required a particular temperature for its phonological affairs such as panicle initiation and exertion, flowering, which may be influenced by the planting dates [26]. Deviation from the planting time may cause incomplete and irregular panicle exertion, and increased spikelet sterility [27] which resulted in yield reduction.

Among yield components, productive tillers are very important because the final yield is mainly a function of the number of panicles bearing tillers per unit area. No. of tillers at maximum tillering stage and productive tillers are higher in July planting. Prevailing low temperature is not favorable for the elongation of the tillers [28], which may also affect the panicle initiation process resulting in low number of panicle per hill leading to low yield, as grain yield is a function of interplay of various yield components such as number of productive tillers, spikelets per panicle and 1000-grain weight [29]. Net returns were directly related to the system productivity and the production cost, which may depend on the price that producer received for the product. Production cost of dry season crops was lower due to its low labour and less land preparation requirement which led to higher net return, B: C ratio and economic efficiency of the system. Singh *et al*. [30] reported that when post-rainy crops were grown after rice in the same field the highest net return was achieved in rice-groundnut, rice-lentil and rice-rapeseed crop combination compared to monocropped rice. According to Rashid *et al*. [31], the highest gross margin was earned in rice-relay mustard-rice cropping pattern was 63% and gross margin in rice-mustard-rice was 60% higher compared to existing rice–rice cropping pattern in Bangladesh. Samant [32] also found that the lowest production efficiency was found in rice-fallow system due to lower yield in rice and maximum land use efficiency was observed in rice-groundnut cropping system followed by rice-brinjal with greater combined yield. Rice-fallow, had given relatively lower yield due to its longer duration with less return [33]. Production cost of the R_S_*-BG/GG/T/R cropping system was highest because first crop cultivation consumes input but did not yield any output resulting in low B:C ratio, but the system is an option to mitigate the risk of weather aberration. According to Davis [34], more diverse cropping systems can use small amounts of inputs as powerful tools with which to tune, rather than drive, agroecosystem performance, while meeting or exceeding the performance of less diverse systems.

## Conclusions

The study amply demonstrated the potentiality of growing atleast two crops in rainfed rice ecosystem utilizing residual soil moisture for higher productivity and profitability of rainfed rice based cropping systems. The system productivity of the systems after inclusion of one dry crop has been enhanced up to 6–6.5 t ha^-1^, which was generally 3.5–5.5 t ha^-1^ when a single crop of rice was grown. Further, it can be concluded that transplanting time significantly affected crop growth, remobilization of photosynthates and yield of rice and following dry season crops mainly due to prevailing weather conditions. Transplanting during July month is most suitable for obtaining better yields of all the rice cultivars, but if transplanting is to be done late, short duration cultivars like Naveen must be chosen for better productivity and profitability. In the dry season, toria is profitable when sown earlier and if sowing is delayed greengram is suitable. Under normal and late sown conditions, R_G_-GG/T/Co/HG and R_N_-GG/T/BG/HG was most productive and profitable systems, respectively, however, in case of weather aberrations profitable cropping systems may not be viable for subsistence of farmers. To overcome this, R_S_*-BG/GG/T/R cropping system may helpful for need based livelihoods. Crop/varietal diversifications help farmers to grow two or more crops/varieties in a year where they could only grow one crop. This combines early maturing varieties of rice with dry season crops like green gram, toria, and blackgram. Because rice can be harvested early, there's time to sow these crops to take advantage of the moisture still left in the soil. This system could impact over 15 million hectares of fallow land in South Asia.

## Supporting information

S1 Appendix Management practices for individual crops grown during the field experiment. (DOCX)

S2 Appendix Input requirements of the individual crops grown during the field experiment. (DOCX)

S3 Appendix Prevailing weather conditions during the period of experimentation in 2012–13 and 2013–14. (DOCX)

## References

[1] Yoshida S. Tropical Climate and Its Influence on Rice. Research Paper Series No.; 20. International Rice Research Institute, Los Baños, Philippines; 1978.

[2] Ramanathan V, Chung C, Kim D, Bettge T, Buja L, Kiehl JT, et al Atmospheric Brown Clouds: Impacts on South Asian Climate and Hydrological Cycle. Proc Nat Acad Sci USA. 2005;102(15): 5326–5333. 10.1073/pnas.0500656102 15749818 PMC552786

[3] Padma Kumari B, Londhe AL, Daniel S, Jadhav B. Observational Evidence of Solar Dimming: Offsetting Surface Warming Over India. Geophys Res Lett. 2007; 34(21):

[4] Wassmann R, Jagadish SVK, Heuer S, Ismail A, Redona E, Serraj R, et al Climate Change Affecting Rice Production: The Physiological and Agronomic Basis for Possible Adaptation Strategies. Adv Agron. 2009;101: 59–122.

[5] Peng S, Huang J, Sheehy JE, Laza RC, Visperas RM, Zhong X, et al Rice yields decline with higher night temperature from global warming. Proc Nat Acad Sci USA. 2004;101(27): 9971–9975. 10.1073/pnas.0403720101 15226500 PMC454199

[6] Hossain M. Rice supply and demand in Asia: a socioeconomic and biophysical analysis In: Teng P.S., et al (Eds.), Applications of Systems Approaches of the Farm and Regional Levels. Kluwer Academic Publishers, Dordrecht, the Netherlands; 1997 pp. 263–279.

[7] Cassman KG, Dobermann A, Stacruz PC, Gines GC, Samson MI, Descalsota JP, et al Soil organic matter and the indigenousnitrogen supply of intensive irrigated rice systems in the tropics. Plant Soil.1996;182: 267–278.

[8] Mujeri MK, Shahana S, Tahrima T, Khondoker C, Haider T. Improving the effectiveness, efficiency and sustainability of fertilizer use in South Asia. The Global Research Capacity Building Program, Policy Research Paper, 8, 2012. pp.389–462.

[9] Masutomi Y, Takahashi K, Harasawa H, Matsuoka Y. Impact assessment of climate change on rice production in Asia in comprehensive consideration of process/parameter uncertainty in general circulation models. Agric Ecosyst Environ. 2009;131(3–4): 281–291.

[10] Kabisch N, Frantzeskaki N, Pauleit S, Naumann S, Davis M, Artmann M, et al Nature-based solutions to climate change mitigation and adaptation in urban areas: perspectives on indicators, knowledge gaps, barriers, and opportunities for action. Ecol Soc. 2016;21(2): 39.

[11] Subbarao GV, Kumar Rao JVDK, Kumar J, Johansen C, Deb UK, Ahmed I, et al Spatial distribution and quantification of rice-fallows in South Asia—potential for legumes. International Crops Research Institute for the Semi-Arid Tropics, Patancheru, Andhra Pradesh, India; 2001 pp. 316.

[12] MoA. Report of Expert Group on Pulses. Department of Agriculture and Cooperation, Ministry of Agriculture, Govt. of India, New Delhi; 2013. pp. 9–10.

[13] Rotter R, Van de Geijn SC. Climate change effects on plant growth, crop yield and livestock. Climate Change.1999;43(4): 651–681.

[14] Xiong J, Ding CQ, Wei GB, Ding YF, Wang SH. Characteristic of dry-matter accumulation and nitrogen- uptake of super-high-yielding early rice in China. Agron J. 2013;105: 1142–1150.

[15] Kar G, Singh R, Verma HN. Alternative cropping strategies for assured and efficient crop production in upland rainfed rice areas of eastern India based on rainfall analysis. Agric. Water Manage.2004;67: 47–62.

[16] Gebrehiwot T, van der Veen A. Farm level adaptation to climate change: The case of farmers in the Ethiopian highlands. Environ Manage.2013; 52: 1–16. 23728486 10.1007/s00267-013-0039-3

[17] Zentner RP, Wall DD, Nagy CN, Smith EG, Young DL, Miller PR, et al Economics of crop diversification and soil tillage opportunities in the Canadian prairies. Agron J. 2002; 94: 216–230.

[18] Chen CB. Farmers’ diversified behavior: An empirical analysis. J Agrotech Econ. 2007; 1: 48–54.

[19] Sun YF, Liang JM, Ye J, Zhu WY. Cultivation of super-high yielding rice plants. China Rice 1995; 5: 38–39.

[20] Nahar K, Hasanuzzaman M, Majumder RR. Effect of low temperature stress in transplanted aman rice varieties mediated by different transplanting dates. Acad J Plant Sci. 2009; 2 (3): 132–138.

[21] Ghosh DC, Singh BP. Crop growth modeling for wetland rice management. Environ. Ecol.1998; 16(2): 446–449.

[22] Ahmadi A, Siuosimardeh A, Zali A. Comparison of storage capacity and photosynthesis matter remobilization and their role in four cultivars of wheat in suitable aggregation and stress conditions. Iranian J Agric Sci. 2004; 35(4): 921–931.

[23] Liu Q, Wu X, Ma J, Chen B, Xin C. Effects of Delaying Transplanting on Agronomic Traits and Grain Yield of Rice under Mechanical Transplantation Pattern. PLoS One. 2015; 10(4), 0123330. 10.1371/journal.pone.0123330 PMC4395310 25875607

[24] Yang J, Peng S, Zhang Z, Wang Z, Visperas RM, Zhu Q. Grain and dry matter yields and partition of assimilate in Japonica/Indica hybrid rice. Crop Sci. 2002;42: 766–777.

[25] Kirchhof G, So HB, Adisarwanto T, Utomo WH, Priyono S, Prastowo B, et al Growth and yield response of grain legumes to different soil management practices after rainfed lowland rice. Soil Till Res. 2000; 56: 51–66.

[26] Yoshida S. Fundamentals of rice crop science. International Rice Research Institute, Los Banos Laguna, Philippines 1981; 267.

[27] Magor NP. A cropping pattern model for rainfed lowland rice in Bangladesh. M.Sc. Ag. Thesis, Faculty of. Agriculture, The University of Sydney, Sydney N.S.W., Australia. 1984; 3–38.

[28] Matsushima S, Tanaka T, Hoshino T. Analysis, of yield-determining process and its application to yield-prediction and culture improvement of lowland rice. LXXV, Temperature effects on tillering in case of leaves and culm, culm-bases and roots being independently treated. Proc Crop Sci Soc Japan. 1966; 34: 478–483.

[29] Hassan G, Khan NU, Khan QN. Effect of transplanting date on the yield and yield components of different rice cultivars under high temperature of D.I. Khan. Science Khyber. 2003;16(2): 129–137.

[30] Singh AK, Chakraborti M, Datta M. Improving rice-based cropping pattern through soil moisture and integrated nutrient management in mid-tropical plain zone of Tripura, India. Rice Sci. 2014; 21(5): 299–304.

[31] Rashid MH, Islam MK, Nasrin RS. Increasing productivity of rice-rice cropping system adopting short duration rice and mustard and relay cropping. International Conference on Environment, Agriculture and Food Sciences (ICEAFS'2012) August 11–12, 2012, Phuket, Thailand, pp 13–16.

[32] Samant TK. Effect of rice based cropping systems and nutrient management practices on yield, economics and soil fertility status. J Agric Forestry & Environ Sci. 2015; 1: 13–16.

[33] Olekar JN, Venkatraman NAD, Kerur NM, Hiremath GM. An economic analysis of rice based cropping sequences. Karnataka J Agric Sci. 2000; 13(4): 897–900.

[34] Davis AS, Hill JD, Chase CA, Johanns AM, Liebman M. Increasing Cropping System Diversity Balances Productivity, Profitability and Environmental Health. PLoS One.2012; 7(10): e47149 10.1371/journal.pone.0047149 23071739 PMC3468434

