# Supplementary material for: Crop and varietal diversification of rainfed rice based cropping systems for higher productivity and profitability in Eastern India
Source: PLoS One. 2017 Apr 24;12(4):e0175709. doi: 10.1371/journal.pone.0175709 (PMC5402987; doi:10.1371/journal.pone.0175709)
Supplement: S1 Appendix — (DOCX) [file pone.0175709.s001.docx]

**Appendix 1** Management practices for individual crops grown during the field experiment

| **Crop** | **Cultivar** | **Seed rate**  **(kg/ha)** | **Spacing**  **(cm)** | **Crop Season** | **No. of irrigation** | **Nutrient rate (kg/ha)** | **Time of fertilizer application** | **No. of weeding** |
| --- | --- | --- | --- | --- | --- | --- | --- | --- |
| Rice | Naveen | 50 | 20x15 | Kharif | 0 | 80:40:40 | ½ N + full P and K as basal; ¼ N at AT and ¼ N at PI | 1 |
|  | Gayatri | 60 | 20x15 | Kharif | 0 | 80:40:40 |  | 1 |
|  | Swarna | 60 | 20x15 | Kharif | 0 | 80:40:40 |  | 1 |
|  | Annada | 50 | 20x15 | October | 4 | 60:20:20 |  | 2 |
| Green gram | T-150 | 25 | 30x10 | Rabi | 2 | 20:40:40 | Basal | 1 |
| Horse gram | S-164 | 10 | 30x10 | Rabi | 2 | 20:40:40 | Basal | 1 |
| Coriander | Swati | 30 | 20x10 | Rabi | 2 | 60:30:0 | Basal | 1 |
| Black gram | Sarla | 15 | 30x10 | Rabi | 2 | 20:40:40 | Basal | 1 |
| Toria | M-27 | 5 | 30x15 | Rabi | 2 | 60:40:40 | ½ N + full P and K as basal  and ½ N at 45 DAS | 1 |
