# Supplementary material for: Crop and varietal diversification of rainfed rice based cropping systems for higher productivity and profitability in Eastern India
Source: PLoS One. 2017 Apr 24;12(4):e0175709. doi: 10.1371/journal.pone.0175709 (PMC5402987; doi:10.1371/journal.pone.0175709)
Supplement: S2 Appendix — (DOCX) [file pone.0175709.s002.docx]

**Appendix 2** Input requirements of the individual crops grown during the field experiment

| **Item** | **Kharif rice** | | | | | **Green gram** | **Horse gram** | **Toria** | **Coriander** | **Black gram** |
| --- | --- | --- | --- | --- | --- | --- | --- | --- | --- | --- |
|  | **Naveen** | **Gayatri** | **Swarna** | **Annada** | |  |  |  |  |  |
| Fertilizer (kg/ha) | | | | | |  | | | | |
| N | 80 | 80 | 80 | 60 | | 20 | 20 | 60 | 60 | 20 |
| P | 17 | 17 | 17 | 0 | | 17 | 17 | 17 | 13 | 17 |
| K | 33 | 33 | 33 | 0 | | 33 | 33 | 33 | 0 | 33 |
| Seed (kg/ha) | 50 | 60 | 60 | 50 | | 25 | 10 | 5 | 30 | 15 |
| Pesticide(kg/ha) | 1.0 | 1.0 | 1.0 | - | | - | - | 1.5 | - | - |
| Irrigation(mm/ha) | 0 | 0 | 0 | 200 | | 100 | 100 | 100 | 100 | 100 |
| Diesel (L/ha) | 28 | 28 | 28 | 23 | | 10 | 10 | 15 | 12 | 10 |
| Tractor (H/ha) | 10 | 10 | 10 | 10 | | 6 | 6 | 8 | 8 | 6 |
| Labour before harvest (8-hr/day/ha) | | | | | | | | | | |
| Men | 42 | 42 | 42 | 38 | 12 | | 12 | 20 | 15 | 12 |
| Women | 70 | 79 | 76 | 54 | 18 | | 18 | 32 | 20 | 18 |
| Labour for harvest and processing (8-hr/day/ha) | | | | | | | | | | |
| Men | 32 | 32 | 32 | 24 | 12 | | 7 | 19 | 7 | 7 |
| Women | 48 | 48 | 48 | 36 | 18 | | 18 | 28 | 18 | 18 |
