# Supplementary material for: Crop and varietal diversification of rainfed rice based cropping systems for higher productivity and profitability in Eastern India
Source: PLoS One. 2017 Apr 24;12(4):e0175709. doi: 10.1371/journal.pone.0175709 (PMC5402987; doi:10.1371/journal.pone.0175709)
Supplement: S3 Appendix — (DOCX) [file pone.0175709.s003.docx]

**Appendix 3** Prevailing weather conditions during the period of experimentation in 2012-13 and 2013-14

| **Month** | **Maximum temperature (°C)** | | **Minimum temperature(°C)** | | **Evaporation (mm)** | | **Sunshine hours** | |
| --- | --- | --- | --- | --- | --- | --- | --- | --- |
|  | **2012-13** | **2013-14** | **2012-13** | **2013-14** | **2012-13** | **2013-14** | **2012-13** | **2013-14** |
| May | 36.80 | 35.80 | 27.10 | 25.04 | 7.50 | 6.10 | 5.80 | 5.80 |
| Jun | 35.65 | 32.98 | 27.02 | 25.95 | 3.23 | 4.76 | 5.60 | 4.63 |
| Jul | 30.51 | 30.17 | 25.50 | 25.95 | 2.42 | 2.90 | 3.91 | 3.86 |
| Aug | 30.41 | 31.17 | 25.64 | 26.48 | 3.26 | 4.15 | 3.92 | 4.14 |
| Sep | 31.41 | 30.83 | 25.85 | 25.79 | 3.92 | 4.02 | 4.07 | 4.16 |
| Oct | 30.66 | 29.61 | 22.98 | 24.49 | 6.55 | 4.58 | 3.90 | 3.89 |
| Nov | 28.86 | 28.43 | 19.23 | 18.69 | 5.96 | 6.71 | 3.68 | 3.90 |
| Dec | 28.14 | 27.33 | 15.08 | 15.04 | 7.74 | 6.86 | 3.74 | 3.67 |
| Jan | 27.07 | 26.94 | 14.86 | 15.62 | 5.80 | 6.04 | 3.66 | 3.69 |
| Feb | 29.49 | 29.09 | 17.58 | 18.03 | 7.09 | 7.50 | 3.90 | 3.43 |
| Mar | 34.77 | 31.97 | 21.64 | 21.90 | 7.42 | 7.28 | 4.34 | 3.89 |
| Apr | 35.59 | 40.13 | 24.05 | 25.10 | 7.76 | 7.85 | 5.34 | 4.99 |
